# Fighting tuberculosis hand in hand: A call to engage communities affected by TB as essential partners in research

**DOI:** 10.1371/journal.pgph.0004437

**Published:** 2025-04-09

**Authors:** N. Venkatesan, L. Faust, R. Lobo, H. Enkh-Amgalan, T. Kunor, Z. Sifumba, S. Rane, K. O’Brien, B. Kumar, C.N. Maimbolwa, M. Mayta, H. Patel, L.M. Huong, P. Heitkamp, S. Huddart, K. Romanowski, M. Hiebert, A.J. Zimmer, E.L. MacLean, G. Caceres-Cardenas, L. Villa Castillo, J. Black, M. Batchu, C.A. Tschampl, E. Rea, T. Campbell, C. Heffernan, R. Long, L. Raithby, A. Daftary, Y. Chorna, A. Zheng, L. Martinez, S. Kulkarni, C.M. Denkinger, M.D.M. Castro, G. Sulis, J. Furin, L. McKenna, M. Frick, R.R. Nathavitharana, C. Oga-Omenka, R. Ananthakrishnan, J. Malar, C. Ugarte-Gil, W. Vandevelde, A.D. Kerkhoff, P. Winarni, G. Fox, T.A. Nguyen, A.K.J. Teo, H.M. Yapa, N.Y. Pham, A. Ratnasingham, S. Bernays, H.D. Trinh, U. Khan, G.G. Alvarez, A. Deluca, M. Nash, O. Rucsineanu, A. Vasiliu, J. Stillo, P. Nahid, M. Pai, J. Johnston, A.D. Harries, J.E. Golub

**Affiliations:** 1 Community Advisory Board, McGill International TB Centre, Montréal, Canada; 2 Stop TB Canada Network, Ottawa, Canada; 3 Faculty of Infectious and Tropical Diseases, London School of Hygiene and Tropical Medicine, London, United Kingdom; 4 Community Delegation, Stop TB Partnership, Geneva, Switzerland; 5 World Health Organization Civil Society Task Force on TB, Geneva, Switzerland,; 6 We Are TB, United States of America; 7 TB Proof, Cape Town, South Africa; 8 Survivors Against TB, Delhi, India; 9 Global Coalition of TB Advocates, Delhi, India; 10 Community Initiative for Tuberculosis, HIV/AIDS and Malaria (CITAM+), Lusaka, Zambia; 11 Asociación de Personas Afectadas Por Tuberculosis (ASPAT) Péru, Lima, Peru; 12 TBpeople Canada, Ottawa, Canada; 13 Community Advisory Board, Vietnam; 14 TB PPM Learning Network, Montréal, Canada; 15 Department of Epidemiology and Biostatistics, University of California San Francisco, San Francisco, California, United States of America; 16 UCSF Center for Tuberculosis, University of California San Francisco, San Francisco, California, United States of America; 17 McGill University, Montréal, Canada; 18 Provincial Tuberculosis Services, British Columbia Centre for Disease Control, Vancouver, Canada; 19 University of Manitoba, Winnipeg, Canada; 20 Department of Epidemiology, Biostatistics and Occupational Health, McGill University, Montréal, Canada; 21 McGill International TB Centre, Montréal, Canada; 22 Faculty of Medicine and Health, The University of Sydney, Sydney, Australia; 23 Instituto de Medicina Tropical Alexander von Humboldt, Universidad Peruana Cayetano Heredia, Lima, Peru; 24 The Heller School for Social Policy and Management, Brandeis University, Waltham, Massachusetts, United States of America; 25 Stop TB United States of America, Atlanta, Georgia, United States of America; 26 Department of Public Health, Nova Southeastern University, Davie, Florida, United States of America; 27 University of Toronto, Toronto, Canada; 28 Toronto Public Health, Toronto, Canada; 29 Tuberculosis Program, Northern Inter-Tribal Health Authority, Prince Albert, Canada; 30 Tuberculosis Program Evaluation and Research Unit, Department of Medicine, College of Health Sciences, University of Alberta, Edmonton, Canada; 31 Results Canada, Ottawa, Canada; 32 School of Global Health and Dahdaleh Institute of Global Health Research, York University, Toronto, Canada; 33 TB Europe Coalition, Utrecht, The Netherlands; 34 Dahdaleh Institute of Global Health Research, York University, Toronto, Canada; 35 Department of Epidemiology, School of Public Health, Boston University, Boston, Massachusetts, United States of America; 36 Department of Global Health, School of Public Health, Boston University, Boston, Massachusetts, United States of America; 37 Section of Infectious Disease, Boston Medical Center, Boston, Massachusetts, United States of America; 38 Department of Infectious Disease and Tropical Medicine, Heidelberg University Hospital, German Center of Infection Research, partner site Heidelberg, Heidelberg, Germany; 39 School of Epidemiology and Public Health, Faculty of Medicine, University of Ottawa, Ottawa, Canada; 40 Clinical Epidemiology Program, Ottawa Hospital Research Institute, Ottawa, Canada; 41 Department of Global Health and Social Medicine, Harvard Medical School, Boston, Massachusetts, United States of America; 42 Treatment Action Group, New York City, New York, United States of America; 43 Beth Israel Deaconess Medical Center & Harvard Medical School, Boston, Massachusetts, United States of America; 44 School of Public Health Sciences, University of Waterloo, Waterloo, Canada; 45 Resource Group for Education and Advocacy for Community Health (REACH), Chennai, India; 46 Stop TB Partnership, Geneva, Switzerland; 47 Department of Epidemiology, School of Public and Population Health, University of Texas Medical Branch, Galveston, Texas, United States of America; 48 Global Network of People living with HIV (GNP+), Cape Town, South Africa; 49 Division of HIV, Infectious Diseases and Global Medicine, Zuckerberg San Francisco General Hospital and Trauma Center, University of California San Francisco, San Francisco, California, United States of America; 50 Pejuang Tangguh TB RO Jakarta (PETA), Jakarta, Indonesia; 51 Global TB Community Advisory Board, New York City, New York, United States of America; 52 Department of Respiratory Medicine, Royal Prince Alfred Hospital, Camperdown, New South Wales, Australia; 53 University of Sydney Vietnam Institute, Vietnam; 54 Saw Swee Hock School of Public Health, National University of Singapore and National University Health System, Singapore; 55 Department of Infectious Diseases, Westmead Hospital, Sydney, New South Wales, Australia; 56 IRD (Interactive Research & Development) Global, Singapore; 57 Department of Medicine, The Ottawa Hospital and University of Ottawa, Ottawa, Ontario, Canada; 58 Center for Tuberculosis Research, Department of Medicine, Johns Hopkins University, Baltimore, Maryland, United States of America; 59 Moldova National Association of Tuberculosis Patients “SMIT” (Society of Moldova, against Tuberculosis), Balti, Moldova; 60 Global TB Program, Department of Pediatrics, Baylor College of Medicine, Houston, Texas, United States of America; 61 Department of Anthropology, Wayne State University, Detroit, Michigan, United States of America; 62 Department of Global and Public Health, McGill University, Montréal, Canada; 63 University of British Columbia, Vancouver, Canada; 64 International Union Against Tuberculosis and Lung Disease, Paris, France; King's College London, UNITED KINGDOM OF GREAT BRITAIN AND NORTHERN IRELAND

## Abstract

Tuberculosis (TB) is an infectious disease closely intertwined with stigma, discrimination, and the social determinants of health. Communities of people affected by TB are experts in their care pathways, but the TB field continues to fall short of meaningfully engaging communities in TB research. This is a missed opportunity to improve the quality, relevance, person-centeredness, positive impact, and sustainability of TB research outputs. We acknowledge the important progress that has been made to date regarding community engagement in TB, but emphasize persisting barriers to meaningful engagement, and the urgent need for updated and comprehensive TB-specific standards for such engagement in research. We highlight that core components of these standards should include the mobilisation of communities affected by TB, bilateral training in community engagement (for researchers and communities), as well as ensuring appropriate remuneration, representation of priority groups, and the use of non-stigmatising language in the engagement process. In addition, to meaningfully incorporate the experiences and expertise of communities affected by TB, their engagement in the research process should occur as early as possible, ideally before research priorities and directions are set, and the scope of the research should encompass questions and outputs relevant to the community. Further, knowledge-sharing between researchers and the community should be ensured, not only of the research outputs but also regarding the engagement process itself, so that lessons learned can be carried forward. Lastly, the sustainability of community engagement processes (whether within institutions or projects) should be ensured, including through adequate funding for such engagement and the training, community mobilisation and relationship-building that this requires.

“Take urgent action to combat the TB epidemic not just medically but also socially and culturally. …[Engage TB] survivors and patients in decision-making around prevention, testing, treatment and care solutions. Don’t use stigma as an excuse to avoid engaging [people affected by TB] – those with lived experience know best.”

~ *Stigmatized (2021)*, a memoir by Handaa Enkh-Amgalan

(Author, humanitarian & TB survivor)

## People affected by TB as essential partners in TB research

Despite its devastating global impact, progress on tuberculosis (TB) prevention, diagnosis, and treatment has been slow. This is evidenced by inadequate funding for TB research, the use of a limited century-old vaccine (which primarily prevents severe forms of TB in children, but is ineffective at preventing TB in adults), and difficulties in mobilizing political will with coordinated, powerful advocacy [1]. As a group of individuals affected by TB, researchers, healthcare workers, and advocates, we wish to highlight that the TB field is falling short in another crucial area: the practice of meaningful community engagement in TB research. In this commentary, we emphasize the urgent need for updated and comprehensive TB-specific standards, core outcomes, and best practices for community engagement in research and collectively call for their immediate development.

A note on terminology used going forward, for the purposes of this piece, “people affected by TB” refers to people with TB, TB survivors, and their immediate caregivers. The term “communities” refers to groups of people affected by TB that share an experience (i.e., their TB journey) or geographic location. “TB research” refers to the generation of new knowledge regarding TB and how it is applied to TB care. Lastly, “community engagement”, as defined by the World Health Organization (WHO), refers to the process of developing meaningful relationships amongst all stakeholders, to facilitate working together towards optimal health outcomes [2].

Communities of people affected by TB are experts in their care pathways. This includes their journeys through symptom onset, initial care seeking, diagnosis, treatment initiation, treatment completion, and post-TB sequelae, as well as the obstacles they encounter throughout this journey, including stigma, barriers to accessing care, delays in diagnosis, long and difficult treatment regimens, and impacts on long-term health and well-being after TB treatment completion. Consequently, it is evident that community engagement is critical for health research to reflect the context- and setting-specific needs, preferences, and challenges faced by people affected by TB, who have the clearest understanding of these challenges and how best to address them. [3,4] Given that TB is a disease tightly intertwined with stigma, discrimination, and the social determinants of health,[5,6] the knowledge and experiences of people affected by TB are even more crucial to informing strategies to reach those who are currently not well served by or able to access quality TB services. Therefore, their voices should be central to guiding TB research. Despite this being obvious (and repeatedly stated by advocacy groups such as those highlighted below), we have not yet moved from this recognition to meaningful action on community engagement.

As noted by members of the India-based advocacy group Survivors Against TB, metrics and definitions of ‘success’ in TB, including regarding favourable outcomes and quality of care, must also be guided by communities of people affected by TB,[7] whose view of quality care looks beyond the milestones and metrics used in TB programs and quantitative research. For example, a recent Global TB Community Advisory Board (CAB) position paper highlights that the evaluation of new TB regimens should take into account endpoints beyond efficacy that are important to people with TB and more comprehensively capture the treatment experience. These include factors such as regimen tolerability, time spent engaging with the healthcare system, and the overall day-to-day impact of treatment on a person’s quality of life (which is not limited to the impact of specific adverse events) [8,9]. In addition to facilitating the use of person-centered metrics, engaging communities at early stages in the research (i.e., priority-setting and design stages) will allow for early identification of barriers to participation in research, as well as promote the inclusion of groups often omitted from participation, such as children and people who are pregnant/breastfeeding [10,11]. This, in turn, will facilitate early advocacy, ensure product development is in line with community needs (as opposed to simply fulfilling a research agenda), and enable innovations to be made available to communities more rapidly and equitably.

Multiple TB advocacy groups have affirmed that community engagement is central to a human-rights-based TB response [12,13]. Individual calls for community engagement in TB research have also been clear. At the end of her powerful memoir, *Stigmatized*, humanitarian and TB survivor Handaa Enkh-Amgalan calls on TB researchers and healthcare providers to proactively engage people affected by TB in their work [14,15]. TB advocates have also underlined that people affected by TB are a “valuable yet heavily underutilized resource” in the fight to end TB, urging that “policy makers, researchers and implementers must listen to affected communities” if we aim to meet TB elimination goals [16]. In her aptly-named op-ed, *Nothing about us, without us*, journalist, TB survivor, and co-lead author of this paper, Nandita Venkatesan calls out the lack of community engagement in the TB field by healthcare providers and academics not only as an ethical and moral failing, but as a hindrance to TB elimination [3].

Indigenous communities have also long called for more person-centered and community-led TB research, recognizing that knowledge is held by the community, and therefore community engagement – and joint authorship with community members – is an obvious and critical prerequisite in knowledge production [17]. Meaningful community engagement in research supports self-determination and data sovereignty, for example, through the co-development of data governance agreements in equal partnership between researchers and the community, [18] or through the use of research processes based on Indigenous values and conceptualisations of equal partnership [19]. In addition, examples from Indigenous health research have shown that integrating the community in the development of research directions and goals, as well as in all other steps in the research process is critical, from proposal submission to budget development, to data analysis, and finally, return of the findings and study implications to the community, so that research outputs may have a lasting impact [20]. It should be noted, however, that such engagement requires that researchers coming from outside the communities with whom they work receive training in cultural humility, including understanding the social, historical and geographical contexts in which they are working [21].

The human right to “share in scientific advancement and its benefits” is enshrined in international law (specifically in Article 27 of the Universal Declaration of Human rights and further elaborated on in Article 15 of the International Covenant on Economic, Social and Cultural Rights [ICESCR]) [22]. The ‘right to science,’ as it is known, includes the right to “take part in scientific progress and in decisions concerning its direction.” Engaging communities in all stages of the research process—from defining research agendas to target product profiles—is required to realize this right.

The Declaration of the Rights of People Affected by TB, [23] published in 2019, further underscores the right of all people affected by TB to participate in, and enjoy the benefits of, scientific progress. Critically, a recent update from the *Lancet* Commission on Tuberculosis also re-affirms the importance of empowering people affected by TB to lead in “defining and leading the global tuberculosis agenda.” [24] This was further emphasized in the political declaration of the 2023 United Nations High-Level Meeting (UN HLM) on TB, where it was recognized that meaningful engagement of communities affected by tuberculosis is “vital to improve access to TB prevention and care” and “contributes to the promotion and protection of the human rights of people affected by TB.”[25] It follows that community engagement in research, which informs the global TB response, is a critical step. Especially in light of COVID-19-related disruptions to TB programs, harnessing the knowledge and experiences of people affected by TB will be more critical than ever if we are to regain lost ground in TB elimination [26].

In failing to engage people affected by TB in developing novel interventions, strategies and research questions, and interpreting data, we fail to overcome foreseeable barriers to research and implementation, and to maximize the impact of the research. We believe we will not reach the millions of people with TB that go undiagnosed globally each year, holistically treat people with TB, nor prevent and manage post-TB sequalae, unless we undertake research that is person-centred and focused on locally relevant barriers to TB care [4]. This in turn requires the meaningful engagement of people affected by TB and their families, who have the clearest understanding of these barriers and, consequently, of practical solutions.

## Progress on community engagement in TB research

### Existing guidelines for community engagement

While acknowledging that important gaps persist, we recognize the collective efforts that have resulted in significant progress on community engagement in TB,[27] including affirmations of the need for community engagement (described above), and global guidelines to help action these calls (described below).

Good participatory practice (GPP) guidelines for TB trials [28] were published in 2012 (adapted from GPP guidelines for HIV) [29]. Although these guidelines highlight principles relevant to community engagement in TB (such as respect and mutual understanding), they are highly focused on clinical trials and do not encompass broader research, such as implementation and operational research, in which community engagement is also critical. Moreover, the guidelines, which are now over 10 years old, focus on broadly-defined stakeholder engagement, encompassing all stakeholders in TB trials (including not only trial participants and affected communities, but also non-governmental organisations, the media, and public and private sector research partners). Consequently, up-to-date standards of practice [30] specific to the relationship between TB researchers and communities affected by TB are needed [26]. TB-specific tools to facilitate such engagement are available, and their use should be incorporated into these standards of practice. For example, the Stop TB Partnership’s Words Matter Language Guide (the 2^nd^ edition of which was published in 2022), [31] which aims to promote the use of non-stigmatizing language in the TB field, should be used throughout the TB research process, including during community engagement, and should be adapted to local languages. Academic journals should play a role in supporting the use of such tools, and in particular the use of non-stigmatising language, by making it a requirement for publication where applicable.

Most recently, efforts towards meaningful community engagement in TB have been driven forward by the launch of the WHO guidance on engagement of communities and civil society to end TB, which marks an important development in the prioritisation of this work by global agencies. While we welcome this critical guidance, we point out that it is focused primarily on community engagement in public sector TB care (within TB programs and health systems). Although the guidelines mention the need for community engagement in research – particularly in implementation and operational research, and that such engagement will facilitate better implementation of locally relevant solutions [32] – specific guidelines or standards for community engagement in research are not provided. In addition, the focus on engagement of communities within TB programs does not encompass community engagement in a wider sense, for example in community-wide active case-finding initiatives, which involve communities as a whole, not limited to those who have TB or know they have TB and are already engaging with TB programs.

Therefore, in existing guidelines, an important gap remains regarding standards for and consensus on meaningful engagement of communities, not just in the TB response itself but at earlier stages, including in the design, conduct and dissemination of research. Such research-specific guidelines regarding community engagement - which we call for in this work - would facilitate accountability among researchers and their institutions regarding meaningful community engagement, equip researchers to carry out community engagement more effectively, and allow a “designing for dissemination” approach,[33] enhancing the relevance, uptake, sustainability, and positive impact of interventions in communities. It should be noted, however, that such guidelines are simply a starting point for efforts towards meaningful engagement, and should be adapted to work in specific contexts.

### Community engagement in practice

Civil society groups are key facilitators and supporters of community engagement in research. For example, Treatment Action Group (TAG), who were central in pushing for the funding, development and accessibility of HIV therapies through large-scale community engagement in research and policymaking [1] have subsequently led successful community engagement efforts in TB,[34] most notably through the establishment of two community advisory boards, the Global TB Community Advisory Board (Global TB CAB), and the Community Research Advisors Group (CRAG). These groups have highlighted key shortcomings in trials of TB treatments, preventive therapy, vaccines, and diagnostic tools, including the lack of plans to disseminate results or make the evaluated interventions accessible to affected communities following trial completion [35]. Although community engagement in TB research is not yet widely normalised, since its formation, the Global TB CAB has made significant headway in demonstrating its importance and impact [34,36]. The WHO established a civil society task force for TB in 2018, [37] with the aim of incorporating civil society input on WHO TB guidelines, End TB Strategy implementation, and actioning the calls in the political declaration of the UN HLM on TB. Furthermore, the voices of people affected by TB are now more frequently heard at TB conferences, [38] and academic TB centres are setting up TB CABs [39,40] alongside site- and country-specific CABs. Community groups at the sub-national and national levels, such as those involved in the EndTB trials in Pakistan, [41] have demonstrated the importance of meaningful community participation in TB research for subsequent knowledge dissemination and stigma reduction. Further, the multi-country STREAM study [42] investigating shorter regimens for multidrug-resistant TB included a robust community engagement plan, and demonstrated various benefits of community engagement in research, including the finding that CABs can serve as mechanisms for improving research literacy and awareness of TB in communities as a whole [43]. Lessons learned from this engagement process were also highlighted, including the importance of diverse representation among CAB members, the need for adequate funding, clear procedures for engagement, and guidelines outlining CABs’ responsibilities and how they should operate [43].

Funding mechanisms continue to support the critical work of civil society groups, particularly with respect to community engagement in TB research, including the Challenge Facility for Civil Society, [44] TB REACH, Unitaid RFP, and USAID SMART4TB CABs [45]. These types of funding have been critical to establishing community-engaged research collaborations, including stigma intervention co-development studies led by TB survivor peer research associates from TB Proof, which have highlighted the value of TB survivors in designing and delivering interventions informed by their lived experiences [46]. Increased and sustained funding is needed to support such efforts, as is the assurance that such resources directly reach affected communities (for example through the inclusion of specific line items in grants for dedicated funding to be provided to community members involved in CABs).

Increasingly, and especially as it relates to Indigenous health research, national granting agencies (for example, in Canada [47] and Australia [48]), are requiring evidence of meaningful community engagement in the their Requests for Applications. Beyond research funders, organisations, academic institutions, and civil society, National TB Programs are also beginning to take action on community engagement in research. For example, the Inuit TB Elimination Framework (Canada)—developed by the national Inuit organisation, Inuit Tapiriit Kanatami—has a strong focus on community engagement, and on tackling the social determinants of health as core elements of TB elimination efforts, and importantly, also includes a call for Inuit-led research across all aspects of TB prevention and care [49].

## Persisting gaps and obstacles for community engagement

While community engagement in research for HIV has been enormously effective in advancing economic, social, and cultural rights, a similarly large-scale, rights-based, participatory, and people-centered movement in TB research is still lacking. Reasons for the differences in the HIV and TB advocacy movements have been extensively explored, and include the strengthening of the HIV movement as part of a larger gay rights movement. Furthermore, the high level of public attention to HIV upon its emergence facilitated the growth of awareness and advocacy campaigns well prior to the arrival of antiretroviral drugs (ARVs), meaning that by the time ARVs were available, a strong and informed community of advocates was already in place and demanding people-centred care. A similar sense of urgency has been absent in TB, in part due to the fact that communities affected by TB are often communities not prioritised by policymakers. Lastly, people living with HIV are part of an enduring community (given the lack of a cure), whilst people affected by TB may no longer identify as such after successful treatment completion and resolution of symptoms [50]. However, with well over 150 million TB survivors at risk of increased morbidity and mortality due to post-TB lung disease and other post-TB sequelae, [51] such an enduring community is starting to emerge, as TB survivor groups are growing in number globally. Nevertheless, the lack of a large-scale move towards community engagement as the norm in TB is a lost opportunity for improving the quality, relevance, and impact of TB research. As a whole, the field of TB research has yet to commit the required resources toward making meaningful community engagement a standard practice. An accountability report by communities affected by TB and civil society calls out the deadly divide between commitments pledged at the first UN HLM on TB, versus the lack of progress on creating a community-engaged TB response [52].

### The unequal distribution of power in knowledge generation

Current efforts toward meaningful community engagement in research continue to be limited by several shortcomings. First is the perception of people affected by TB as “non-experts” or simply as beneficiaries of TB research. This means that community engagement is often only recognized as having moral value, rather than as a process that is critical to the quality and utility of the work being conducted. In the interest of realizing the right to science, the ICESCR cautions against this siloed view of scientists versus the community and underlines that both have critical roles in the scientific process [53]. For example, a global review of community engagement in TB prevention and care interventions has shown that community participation facilitates implementation of these interventions within TB programs, [54] and addresses barriers to care identified by the affected communities themselves. Yet, academia has a tendency to operate in silos, and lacks equitable and transparent researcher-community partnerships. This means that researchers may not see community engagement as within their purview, or fail to acknowledge the knowledge held by communities. Even when researchers do appreciate the value of community engagement, the types of partnerships with community-led organizations that underpin successful community engagement in research are often not in place, making routine and meaningful engagement challenging. Often, the way in which academic internal review boards (IRBs) operate add to this challenge, as, in the absence of a pre-existing CAB, it can be difficult to conduct wider community consultation without first setting up such consultation as a project in its own right. In practice, therefore, it is clear that gaps remain in realizing the ‘right to science.’ As evidenced by the involvement of CABs such as CRAG and the Global TB CAB in advocating for the inclusion of key populations in TB trials and the accessibility of trial products, ensuring access to the “benefits of science” for affected communities necessitates their participation in the scientific process [35]. Moving beyond a siloed approach to research and allowing for greater flexibility on the part of IRBs and other institutional mechanisms is therefore crucial to fully recognizing the agency of affected communities and their role in research and facilitating community engagement, ownership and autonomy.

### Prioritising outputs over processes

Furthermore, historically, the output-oriented, rather than process-oriented, nature of academia has limited the value attributed to long-term investments in community-building for effective community-centered research. Similarly, the lack of prioritization and incentivization of community engagement, particularly in quantitative research disciplines, means that opportunities for training researchers in meaningfully seeking and incorporating community perspectives into research are lacking. Although community engagement in research on other infectious diseases has been impactful, [55,56] such engagement is not systematic. For example, it is not yet a requirement for WHO Target Product Profile (TPP) development, which is itself a standardised process, meaning that the integration of community engagement in such processes would be a significant step towards systematising and engraining the practice of community engagement in TB.

Another challenge to achieving effective and respectful community engagement is inherent in the nature of research conduct and funding, where budgets, milestones, grant cycles, and the rush to publish, may mean that researchers engage people affected by TB only at later stages of research, when research questions and priorities have already been set, or that researchers consider such engagement but cannot follow through due to external time constraints [57]. This is a missed opportunity to actualize the ‘right to science’ mentioned previously, which refers not only to the right to benefit from scientific progress, but also to the right to *participate* in it, and to take part in “decisions concerning its direction.” [53] Failure to engage communities at early stages in the research process (i.e., before a grant application is submitted) can also mean that research, once published, is not optimally translated into practice, and valuable opportunities for knowledge translation are lost. This hinders genuinely incorporating the insights of people affected by TB, and truly centering research around their priorities. In particular, a further gap in community engagement early in the research process is the current lack of community participation in IRBs, before research projects are even approved. This is especially relevant for those making risk vs. benefit decisions regarding the inclusion or exclusion of key populations, such as people who are pregnant/breastfeeding in proposed research studies [10,11].

### TB stigma as a barrier to engagement

Due to the unique and persisting experiences of stigma and discrimination faced by people affected by TB, [6] efforts towards community engagement may continue to be particularly challenging in TB, compared to other research areas. In addition, people affected by TB may want to leave their often difficult and traumatic experiences with TB behind them once they complete treatment, making it more challenging to engage people with lived experiences of TB in research, as opposed to, for example, people living with HIV or other chronic conditions, who continue to engage with the healthcare system.

### The challenge of community representation

A further obstacle to meaningful community engagement is the tendency to consider a “community” as one homogeneous group, which hinders seeking out the unique perspectives of sub-groups with different experiences, or from diverse regions or cultures. As efforts towards community engagement grow in certain priority populations, such as Indigenous communities, [49] similar efforts are needed across all key populations, including people affected by TB who are incarcerated, living with HIV, under-housed, migrants and refugees, and people who use drugs [4]. The unique experiences of people affected by TB in each of these populations are critical, and important nuances across and within different populations can only be gleaned and reflected in research through meaningfully engagement with members of these communities. In addition, answering the question of who speaks for or best represents a particular community and its interests remains an important challenge that requires careful consideration, as do ways in which to integrate perspectives and priorities from different voices within a community.

### The lack of a framework for meaningful community engagement

Lastly, a key barrier to improving the extent and nature of engagement of communities affected by TB in research – and perhaps the most immediately amenable to improvement – is the lack of a framework for how to carry out meaningful community engagement in TB research, and the resources and funding required to implement it, co-developed by TB researchers, advocates and people affected by TB.

## Core components of meaningful community engagement

**Fig 1:**
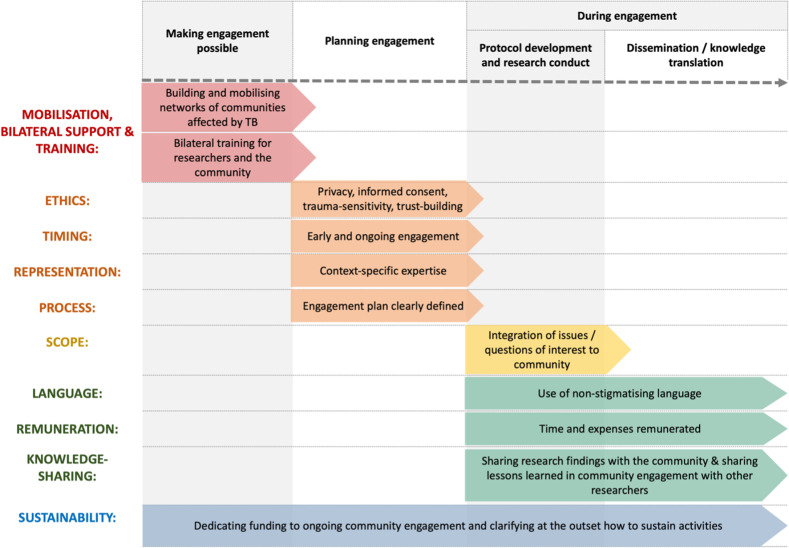
Core components of meaningful community engagement in TB research.

Drawing on our collective experiences, and led by people affected by TB, we are therefore undertaking the co-development of a set of core components of meaningful community engagement (listed below, and summarised in **Fig 1**), as a first step towards improving the practice of community engagement in TB research and making such engagement standard practice in TB.

1) **Mobilisation of communities, bilateral support and training:** For effective community engagement to be possible, TB survivors and advocates must build and mobilise networks of people affected by TB as a critical first step. Subsequently, to facilitate meaningful engagement, resources and training should be offered to people affected by TB who serve on CABs and are interested in further developing their research literacy and associated skills. This will allow fuller CAB participation, by supporting and empowering its members to voice their perspectives, knowledge, and concerns, as well as develop leaders who can then support and train future members. However, this capacity building and skills development should go both ways - apart from community members being supported in increasing their scientific literacy, researchers themselves should also be supported to improve their ability to engage and communicate with community members effectively and respectfully. This includes improving their cultural humility, especially if researchers are not from the region or culture in which they are conducting research. Examples of this include research on TB among and with Indigenous communities [21]. Best practices for community engagement in research should therefore be part of research-based graduate program curricula so that trainees can incorporate them into their work from the outset.
2) **Ethics:** The ethical engagement of communities affected by TB requires particular attention to questions of privacy, informed consent, trauma sensitivity, and appropriateness of processes and compensation to the local context. Firstly, agreeing to share their experiences with TB does not necessarily mean an individual consents to disclosing their TB status - privacy in these situations must be ensured. Furthermore, people affected by TB engaged in research should be fully informed about the risks and benefits of their engagement, and the process of engagement should be sensitive to and respectful of the lived experiences of people affected by TB (e.g., refer to point 7 on non-stigmatizing language). Moreover, given that the research should ultimately be in the service of the community, consent should be sought on an ongoing basis for continued participation in new projects, paying particular attention to research fidelity (i.e., ensuring that data collected for the purpose of answering a particular question is not used to answer other questions to which the community did not agree). To this end, efforts to build trust and minimise power imbalances between academic institutions and the community are needed. Lastly, community representation on research ethics boards would be valuable, or, where not possible, ethics boards should encourage community engagement to be part of submitted protocols.
3) **Timing:** For all types of research (from clinical to implementation studies), engagement as early as possible in the research process is recommended (i.e., in the conceptualisation phase), so that input can be meaningfully incorporated and guide project directions. Ideally, this should involve providing dedicated resources and support for communities to identify and shape their own research priorities and approaches. If possible, engagement should be ongoing (rather than a one-off occurrence) and cover all stages of the research process, from inclusion of people affected by TB in developing grant proposals to being co-authors in publications. However, how often and in what way(s) people affected by TB are engaged should be determined by and adapted to individual preferences. TB centres and research groups should provide the required resources (both monetary and structural) for supporting ongoing engagement, in the form of community advisory groups.
4) **Representation:** All people affected by TB have unique experiences. Therefore, efforts should be made to ensure that insight is sought from people who can best provide context-specific expertise relevant to the setting in which the research is being conducted. In TB, the experiences of key populations such as people who are incarcerated, people living with HIV, migrants and refugees, Indigenous communities, people who are under-housed, people who use drugs, people who are pregnant, children and adolescents and their parents, the elderly, people with disabilities and LGBTQIA+ communities, among others, are unique. Equally important are the perspectives and challenges of those who never reach TB services and are never diagnosed. Efforts to ensure representation from these groups are critical and require specific attention as to how engagement in research can be made practically accessible to these groups. Moreover, these groups are often stigmatized within broader communities, thus close attention should be given to exactly who is considered by a community to best represent them and their interests.
5) **Process:** The goals of engaging people affected by TB in a specific project should be clearly articulated, as should the ways in which input will be sought. Ideally, an engagement plan or Standard Operating Procedure (SOP) should be co-developed with people affected by TB, to outline the engagement process, the associated expectations, and their relationships with researchers, donors, and other stakeholders involved. In addition, the process of engagement (e.g., building a community advisory board, and reaching and engaging its members), should be guided by a flexible framework, and carried out in an accessible, equitable and representative manner, particularly in relation to ensuring engagement of the various key populations highlighted above, as applicable to the study context. Such transparent processes for engagement will also facilitate the evaluation and reporting of engagement (for future knowledge-sharing, see point 9) and help address competition between TB community organisations for representation on scientific projects.
6) **Scope:** For community engagement to be truly meaningful, issues of broader interest to communities should be discussed and incorporated in research projects, rather than dismissed as out-of-scope for a particular study. For example, although many clinical trials in TB do have community advisory boards, other types of studies (e.g., observational or pilot studies) have been less likely to involve people affected by TB. Broader community engagement across different study designs should therefore be encouraged, to reduce the conduct of studies and use of outcome measures – often narrowly and specifically defined by researchers – that do not involve questions, interventions, or outcomes of importance to communities.
7) **Language:** The use of non-stigmatizing language (see the Stop TB Partnership Language Guide)^31^ is a must, not only in communication with the people affected by TB who are being engaged in the research, but in all research outputs [58]. In addition, resources and documents shared with people affected by TB should be made available in lay language or with explanations of technical terminology, as well as in languages other than English where relevant to the local context [43]. Lastly, translation into local languages is also critical for knowledge-sharing of research findings back to the community (see point 9) and therefore applies not only to materials shared with communities during the research and community engagement process, but also to final research publications (at the very least, abstracts).
8) **Remuneration:** CABs and their members should have designated funding to run community engagement activities within a robust engagement plan, and, when engaged individually, people affected by TB should be appropriately remunerated for sharing their time and expertise with researchers. In addition, expenses related to the attendance of people affected by TB at TB conferences and events should be covered, and should ideally be waived or paid up-front rather than reimbursed after the event, to remove barriers to participation and challenges with reporting expenses in contexts where cash transactions are common. Non-financial barriers to participation, including visa challenges, should also be considered when engaging communities. Influential institutions must lead the way. For example, funding bodies are increasingly calling for community engagement in projects, but often fail to make such engagement feasible/accessible, as administrative barriers to full and equal participation of community members as co-investigators continues to be hindered by administrative barriers.
9) **Knowledge-sharing:** There are two critical avenues for knowledge-sharing in the context of community engagement. The first relates to knowledge-sharing regarding community engagement efforts themselves. Considering that community engagement is a dynamic and evolving process, strategies and best practices for engagement should be continually evaluated and improved. Establishing feedback loops by reflecting on and documenting what works, recognizing areas for improvement, and sharing experiences with the broader research community, and between CABs, will ensure that engagement efforts remain relevant and adaptable. The second relates to knowledge-sharing of research outputs back to the community, particularly via community members engaged in research. With consideration of power dynamics in the researcher-community relationship and ensuring that community experiences are valued,[59] researchers and community members should collectively interpret and discuss outputs, as well as decide on materials and strategies for dissemination that resonate most with and are preferred by communities affected by TB. Beyond dissemination, the translation of research to policy and implementation of evidence-based interventions as well as their subsequent monitoring, should occur in partnership with the community.
10) **Sustainability:** The points above, especially those pertaining to remuneration, reporting, knowledge-sharing, monitoring, and training all raise questions of sustainability. How these processes can be sustained - and funded - should be clarified at the outset. For example, it may be preferrable for mechanisms of community engagement such as CABs to be site or geographically based rather than project based. This is because the limitation of their funding and scope to a particular project is a barrier to sustainability, and any expertise that community members gain may not be put to use in future studies or in training further community members to participate in research.

## Conclusion

This is a consensus statement by people affected by TB as well as TB researchers, clinicians, and advocates, in which we recognize that the meaningful engagement of people affected by TB is the collective responsibility of all those working in TB, and that efforts in this area have hitherto been inadequate. With this statement, we underscore our intention to work together to rectify this, starting with the co-development of guidelines for meaningful community engagement in TB research. Our call for the development of a standardised minimum set of requirements and reporting guidelines for community engagement in research will allow for transparency, accountability and standardization (similar to reporting guidelines used for specific study designs, e.g., the Preferred Reporting Items for Systematic Reviews and Meta-Analyses [PRISMA]). For these guidelines to become standard practice in our field, academic institutions, TB researchers, policymakers, and funders must be accountable for improving the ways in which we conduct person-centered research in TB and ensure that it is both relevant to and meaningful for communities affected by TB. These guidelines however are only a step towards more meaningful community engagement, which should be an ongoing process and continually improved upon. Building, strengthening, and sustaining the partnerships necessary for community engagement is essential to reaching the End TB goals.
